# Editorial for Special Issue “Cancer Biomarkers: Discovery and Applications”

**DOI:** 10.3390/cimb48101092

**Published:** 2026-10-21

**Authors:** Chia-Ching Chen, Hsin-Hung Chen, Po-Ming Chen

**Affiliations:** 1 Department of Emergency Medicine, Chang Bing Show Chwan Memorial Hospital, Changhua 500, Taiwan; medilife.c11g@gmail.com; 2 Department of Medical Education and Research, Kaohsiung Veterans General Hospital, Kaohsiung 813, Taiwan; derekchen@vghks.gov.tw; 3 Research Assistant Center, Show Chwan Memorial Hospital, Changhua 500, Taiwan; 4 Department of Nursing, Central Taiwan University of Science and Technology, Taichung 406, Taiwan

## 1. Introduction

The conceptual landscape of clinical oncology and cancer biology has undergone a profound transformation in recent decades, evolving from static anatomical staging and broad histopathological classification into a dynamic, molecularly stratified discipline. Malignant neoplasms are no longer regarded merely as autonomous clusters of hyperproliferative cells; rather, they are increasingly recognized as intricate, evolving ecosystems governed by genomic instability, non-genetic phenotypic plasticity, and complex metabolic and immunological crosstalk within the surrounding microenvironment [1]. In this modern era of precision medicine, the identification, rigorous biological validation, and clinical translation of robust cancer biomarkers have become the paramount compass directing individualized patient care. Historically, diagnostic and therapeutic frameworks were constrained by single-analyte immunohistochemical assessments performed on isolated tissue biopsies, which frequently failed to capture the spatial and temporal clonal heterogeneity inherent to tumor progression. Today, the biomarker paradigm has expanded dramatically to encompass multidimensional analytes spanning circulating cell-free nucleic acids, tumor-derived nanovesicles, spatial cytogenomic structures, and cellular immune checkpoints. These multifaceted biomarkers are reshaping every tier of oncology, facilitating ultrasensitive multi-cancer early detection, continuous tracking of minimal residual disease (MRD), rational therapeutic target discovery, and preemptive identification of therapy-induced clonal resistance [2,3].

Despite substantial advancements in high-throughput sequencing technologies and molecular diagnostics, formidable knowledge gaps and translational hurdles continue to impede routine clinical implementation. A persistent challenge lies in the biophysical scarcity and rapid clearance of circulating tumor-specific molecules within complex biofluids, where background noise from normal hematopoietic cells frequently obscures trace oncogenic signatures. Furthermore, the tumor microenvironment (TME) represents a notoriously dense and immunosuppressive barrier wherein malignant cells co-opt stromal fibroblasts and myeloid populations to evade immune surveillance and establish therapy-resistant niches [4,5]. Conventional single-parameter assays are inherently insufficient to resolve such spatial complexity or to decipher the dynamic structural rearrangements occurring within the interphase nucleus [6]. Compounding these hurdles is the frequent emergence of acquired drug resistance following standard chemotherapeutic regimens, necessitating rational combination strategies informed by reliable pharmacodynamic and mechanistic biomarkers [7]. This Special Issue of *Current Issues in Molecular Biology*, entitled “Cancer Biomarkers: Discovery and Applications”, was established in order to directly confront these fundamental questions and bridge the divide between exploratory bench discovery and bedside clinical application. Gathering original investigations and authoritative syntheses from international leaders in translational oncology, this collection delivers cutting-edge insights spanning liquid biopsy innovations, ultrasensitive nano-immunoassays, tumor microenvironmental immunogenomics, functional 3D cytogenomics, and biomarker-directed synergistic therapeutics.

## 2. Liquid Biopsy Innovations: ctDNA and Minimal Residual Disease Tracking

Among the most revolutionary frontiers in non-invasive biomarker discovery is the clinical maturation of liquid biopsy, particularly the interrogation of circulating tumor DNA (ctDNA). In a comprehensive and insightful review, Yan, Su, and Wang [8] delineate the extraordinary trajectory of ctDNA from bench innovations to bedside implementations. ctDNA, composed of short, fragmented DNA shed by apoptotic, necrotic, or actively secreting tumor cells into the peripheral circulation, encapsulates real-time genomic, epigenomic, and mutational profiles across primary and metastatic lesions. The authors systematically synthesize the mechanistic release dynamics of ctDNA and provide a rigorous comparative analysis of emerging diagnostic platforms, contrasting the high-precision quantification of digital polymerase chain reaction (dPCR) with the expansive breadth of targeted next-generation sequencing (NGS), whole-exome sequencing, and epigenomic fragmentomics [3,8]. Crucially, Yan and colleagues articulate the clinical indispensability of ctDNA for assessing post-surgical minimal residual disease (MRD) in both solid and hematological malignancies. By detecting trace molecular recurrence months before overt anatomical lesions become visible on standard computed tomography or magnetic resonance imaging, longitudinal ctDNA monitoring provides an unprecedented window for preemptive adjuvant intervention [2]. Furthermore, their review highlights how ctDNA profiling captures emergent subclonal mutations driving secondary therapeutic resistance, thereby offering clinicians an agile tool to adapt targeted regimens dynamically without subjecting vulnerable patients to repetitive, invasive tissue biopsies.

## 3. Nanovesicular and Ultrasensitive Biomarker Assays: Trace Immune Checkpoints

ctDNA continues to redefine molecular genomics in liquid biopsy: the phenotypic and immunological status of tumors is largely mediated by extracellular vesicles that circulate within peripheral blood. In this context, Okita, Arita, Sudo, Yoshimura, and Ito [9] present a landmark technological breakthrough addressing one of the most notorious bottlenecks in biofluid biomarker detection: the quantification of low-abundance immune checkpoint proteins. Programmed death-ligand 1 (PD-L1) expression on tumor-derived small extracellular vesicles (sEVs) plays a pivotal role in mediating systemic immunosuppression by engaging PD-1 receptors on cytotoxic T cells at distant metastatic sites, effectively functioning as a decoy that neutralizes anti-tumor immunity [10,11]. However, because sEV-derived PD-L1 (sEVPD-L1) circulates in minute concentrations, conventional enzyme-linked immunosorbent assays (ELISA) routinely lack the analytical sensitivity required for reliable clinical measurement. To overcome this limitation, Okita et al. engineered an ultrasensitive colorimetric platform termed TN-cyclon™, which couples a sandwich ELISA architecture with thio-NAD cycling enzyme amplification [9]. This innovative method achieved an extraordinary limit of detection (LOD) of 0.172 pg/mL for recombinant PD-L1, exhibiting a 20-fold enhancement in analytical sensitivity compared to standard ELISA kits. Applying this platform to clinical specimens, the authors demonstrated that serum sEVPD-L1 concentrations were significantly elevated in stage IVB lung cancer patients compared to healthy donors, whereas conventional soluble PD-L1 (sPD-L1) failed entirely to differentiate between groups [9]. This pilot study elegantly proves that coupling vesicular fractionation with catalytic enzyme cycling unlocks circulating immune checkpoints as powerful non-invasive surrogate biomarkers, providing an accessible, equipment-light framework to guide patient stratification for immune checkpoint blockade therapy.

## 4. Tumor Microenvironmental Immunogenomics: The SPP1–Macrophage Axis

Parallel to circulating biofluid biomarkers, deciphering the localized transcriptional and cellular architecture of the tumor microenvironment remains fundamental to unraveling disease aggressiveness and therapy failure. This is exemplified in triple-negative breast cancer (TNBC), an intensely aggressive and heterogenous breast cancer subtype characterized by the absence of estrogen receptors, progesterone receptors, and HER2 amplification, leaving patients with limited targeted treatment options and poor clinical outcomes [12]. The transcriptomic interrogation of Gene Set Enrichment Analysis (GSEA), large-scale validation across The Cancer Genome Atlas (TCGA) and Kaplan–Meier plotter datasets, and in-house tissue microarray (TMA) immunohistochemistry revealed marked activation of Toll-like receptor signaling pathways in TNBC tissues relative to normal breast parenchyma. Significantly, the authors identified Secreted Phosphoprotein 1 (SPP1, also known as osteopontin) mRNA as a pivotal biomarker whose elevated expression robustly predicts inferior overall and disease-free survival in TNBC cohorts [12]. Crucially, immune infiltration modeling revealed a strong and specific correlation between SPP1 mRNA overexpression and the infiltration of CD206+ M2-polarized tumor-associated macrophages (TAMs). This discovery converges seamlessly with recent high-impact single-cell and spatial immunogenomic studies demonstrating that SPP1+ TAMs constitute a distinct, pro-tumorigenic myeloid subset that coordinates stromal desmoplasia, excludes CD8+ effector T cells, and drives primary resistance to immunotherapy across diverse solid cancers [4,5]. By linking transcriptomic dysregulation to myeloid remodeling and patient survival, Chen and colleagues establish SPP1 as both a vital prognostic biomarker and a compelling molecular target for immunotherapeutic reprogramming in TNBC.

## 5. Spatial Cytogenomics and 3D Nuclear Architecture: Telomeric Remodeling in Leukemia

Broadening the biomarker concept beyond linear nucleic acid sequences and soluble proteins, Morato de Oliveira, Cruvinel, Ferreira, and Mai [13] introduce a captivating spatial cytogenomic paradigm by investigating the three-dimensional (3D) nuclear and telomeric architecture in leukemia. Chromosomal instability (CIN) and aberrant telomere dynamics are universal hallmarks of neoplastic transformation that drive genomic reorganization, clonal heterogeneity, and disease progression [6,14]. Focusing on chronic myeloid leukemia (CML), which is traditionally characterized by the BCR::ABL1 fusion but frequently develops secondary chromosomal aberrations during blast crisis, Morato de Oliveira and colleagues evaluated the antineoplastic and chromatin-remodeling actions of reversine, a potent small-molecule Aurora kinase inhibitor, in K-562 and MEG-01 CML cell models [13]. Utilizing quantitative fluorescence in situ hybridization (Q-FISH) coupled with the validated TeloView^®^ analytical platform, the investigators conducted single-cell 3D spatial profiling of nuclear telomere numbers, signal intensities (proportional to telomere length), telomeric aggregations, nuclear volumes, and spatial distribution parameters (a/c ratios). Treatment with reversine induced profound dose- and time-dependent apoptotic execution alongside substantial downregulation of AURKA and AURKB transcription. Notably, 3D telomeric profiling unmasked a distinct structural signature characterized by a concurrent reduction in telomere number, aggregates, signal intensity, and total nuclear volume [13]. Rather than representing catastrophic, stochastic genomic decay, these orchestrated spatial modulations reflect a coordinated structural remodeling of the interphase nucleus that marks commitment to programmed cell death. Furthermore, divergent architectural responses between K-562 and MEG-01 cell lines highlighted intrinsic heterogeneity in telomere maintenance pathways. This pioneering work positions 3D nuclear telomere profiling as an innovative functional cytogenomic biomarker capable of quantifying drug-induced chromatin reorganization and monitoring therapeutic efficacy in hematological malignancies.

## 6. Pharmacodynamic Biomarkers and Synergistic Anti-Resistance Strategies

A foundational imperative of biomarker discovery is the rational design of therapeutic combinations that circumvent drug resistance and mitigate systemic toxicity. In non-small-cell lung cancer (NSCLC), platinum-based regimens such as cisplatin remain standard first-line therapies; however, intrinsic or acquired chemoresistance and debilitating toxicities routinely restrict their therapeutic window. Karabat and Tuncer [15] confronted this major oncological dilemma by investigating the chemosensitizing and apoptotic potential of acetyl-11-keto-β-boswellic acid (AKBA), a biologically active pentacyclic triterpenoid isolated from Boswellia serrata, in combination with cisplatin against A549 NSCLC cells. Employing a rigorous methodological matrix combining MTS viability assays, Annexin V-FITC/PI flow cytometry, caspase cascade activation, RT-qPCR, and ELISA, the authors demonstrated that the co-administration of AKBA with cisplatin exerted potent synergistic cytotoxicity, significantly enhancing apoptotic rates compared to either monotherapy alone [15]. Mechanistically, this synergy was orchestrated by the robust transcriptional upregulation of the tumor suppressor p53 and executioner caspase-3, in conjunction with marked suppression of the nuclear factor kappa B (NF-κB) survival pathway. To establish structural and biophysical plausibility, Karabat and Tuncer performed molecular docking and molecular dynamics (MD) simulations, demonstrating that AKBA forms direct, highly stable interactions within the DNA-binding domain of the p53 protein [15]. Given that hyperactive NF-κB signaling and impaired p53 surveillance represent primary drivers of platinum resistance and inflammatory survival in lung cancer [7], this study provides compelling proof-of-concept for biomarker-guided adjuvant strategies. By targeting dual apoptotic and anti-inflammatory nodes, the AKBA–cisplatin combination establishes a rational, dose-sparing framework capable of maximizing antineoplastic efficacy and minimizing off-target chemotherapeutic toxicity.

## 7. Concluding Perspectives and Future Directions

When viewed collectively, the five contributions published in this Special Issue synthesize into an integrated, multidimensional continuum that directly dismantles existing bottlenecks in translational cancer research. Historically, biomarker investigations have suffered from methodological fragmentation: biofluid assays, tissue-based pathology, cytogenetics, and preclinical pharmacology were pursued in mutual isolation. The papers assembled here illustrate how these diverse domains powerfully cross-illuminate one another. First, the technological advances presented by Yan et al. [8] and Okita et al. [9] establish how next-generation liquid biopsies can overcome the biophysical sensitivity barrier. By combining comprehensive ctDNA multi-omics with catalytic enzyme cycling for exosomal checkpoint proteins, liquid biopsies transition from exploratory research tools into precise, real-time monitors of minimal residual disease and therapeutic responsiveness. Second, the work of Chen et al. [12] illustrates the necessity of interrogating the tumor microenvironment beyond tumor-intrinsic genomics, proving that stromal macrophage reprogramming mediated by SPP1 is a critical determinant of immune evasion and clinical survival. Third, Morato de Oliveira et al. [13] elevate biomarker analysis into the realm of three-dimensional nuclear architecture, proving that spatial telomeric organization reflects genomic instability and therapeutic commitment in leukemia. Finally, Karabat and Tuncer [15] demonstrate that elucidating the molecular crosstalk between p53 and NF-κB pathways provides the precise mechanistic foundation required to build synergistic, resistance-bypassing combination regimens. Together, these contributions elevate cancer biomarker research from mere descriptive association to functional, actionable, and clinically impactful application.

Building upon the discoveries and conceptual frameworks presented in this Special Issue, several strategic research directions emerge to guide the future trajectory of cancer biomarker development. First, there is an urgent need to develop multi-modal liquid biopsy platforms that integrate disparate circulating analytes, simultaneously interrogating ctDNA fragmentation and methylation signatures, extracellular vesicle surface proteomes, circulating microRNAs, and circulating tumor cells within a unified diagnostic workflow [2,3]. Such multi-analyte integration will substantially elevate diagnostic specificity and sensitivity, particularly in early-stage asymptomatic malignancies where single-analyte concentrations approach detection limits. Second, future investigations must leverage high-resolution spatial multi-omics and single-cell spatial transcriptomics to dissect the microarchitectural niches of immunosuppression in solid tumors. Elucidating how SPP1+ tumor-associated macrophages physically interact with cancer-associated fibroblasts and dysfunctional T cells will enable the design of precise dual-targeting immunotherapies capable of breaching the desmoplastic stroma [4,5]. Third, the integration of artificial intelligence (AI), computer vision, and machine learning into 3D cytogenomic imaging (such as automated TeloView^®^ platforms) promises to establish standardized, high-throughput metrics of nuclear architecture and chromosomal instability, providing clinicians with rapid functional readouts of drug efficacy and malignant transformation [6,14]. Finally, transitioning these preclinical innovations, including the TN-cyclon™ assay, AKBA–cisplatin combinations, and 3D telomeric remodeling profiles, into rigorous, prospective, multi-center clinical trials will be essential to establish their diagnostic accuracy, regulatory compliance, and clinical utility across diverse patient populations.

In conclusion, the articles curated in this Special Issue vividly demonstrate the vibrancy, scientific rigor, and transformative potential of modern biomarker research. From the non-invasive surveillance of circulating tumor DNA and nanovesicular immune checkpoints to the spatial mapping of tumor microenvironments, 3D nuclear architectures, and biomarker-guided synergistic therapies, these studies illuminate the path forward in personalized oncology. As Guest Editor, I extend my profound appreciation to all contributing authors for sharing their exceptional discoveries, to the dedicated expert reviewers for their rigorous evaluations, and to the editorial staff of *Current Issues in Molecular Biology* for their steadfast commitment to scientific excellence. It is our collective hope that this Special Issue will serve as an inspiring catalyst for future investigations, accelerating the translation of innovative cancer biomarkers from the laboratory bench to the patient bedside and ultimately transforming cancer care.

## Data Availability

No new data were created or analyzed in this study. Data sharing is not applicable to this article.
